# Hospital Construction Competitions

**Published:** 1912-05-11

**Authors:** Ian MacAlister

**Affiliations:** Secretary. Royal Institute of British Architects, 9 Conduit Street, Hanover Square, London, W.


### THE EDITOR'S LETTER-BOX.

### Hospital Construction Competitions.

THE EAST SUSSEX HOSPITAL, HASTINGS.

*To the Editor of* The Hospital.

Sir.—The Council of the Royal Institute of British Architects have had brought to their notice the Report and Note on the above competition, published in your paper of March 2 last, purporting to be written by two Fellows of the Royal Institute, and your editorial comments thereon.

The Council regret that two members of the Royal Institute should have anonymously attacked a fellow-member in the pages of a public print, and that editorial comments should have been made in support of *ex parte* statements.

The Council have, through a committee, carefully investigated the allegations made against the assessor, and they find them to be wholly undeserved.

They trust that you will give this letter as much publicity as was given to the matter complained of.—Faithfully yours,

Ian MacAlister, Secretary.

Royal Institute of British Architects,
9 Conduit Street, Hanover Square, London, W.
May 7.

[We gladly publish the above letter. We must, however, repeat the matured conviction which experience has brought us—*i.e.* "One practical reform which the architectural profession should insist upon, so far as the Royal Institute of British Architects is concerned, is the selection of a panel of experienced architects, competent on their merits, to act as assessors. Another is that the selection should never be left to the decision of one assessor, whoever he may be, for the reason that a modern hospital is many-sided, and that no architect by himself has the knowledge to enable him to judge which out of many plans, so far as the administration and requirements of a modern hospital are concerned, is in fact the best. An architect may, if he is conscientious, knowledgeable, experienced and thorough, rightly be called upon to decide most points concerned with buildings, as such, which appertain to the professional work of an architect. For all the rest it is essential that there shall be associated with him a competent hospital expert who is admittedly an authority on the many technical and administrative matters which must materially affect a just and satisfactory decision as to the respective merits of a number of plans for a new hospital submitted in competition."—Ed., The Hospital.]

